# ﻿Three nodulose-spored *Inocybe* (Agaricales, Basidiomycota) species discovered from Motuo, Southwest China

**DOI:** 10.3897/mycokeys.122.163942

**Published:** 2025-09-11

**Authors:** An-Hong Zhu, Yan-Ru Xu, Hai-Xia Ma, Wen-Jie Yu

**Affiliations:** 1 Haikou Key Laboratory for Conservation and Utilization of Edible and Medicinal Fungal Germplasm Resources, Hainan Key Laboratory of Tropical Microbe Resources, Institute of Tropical Bioscience and Biotechnology, Chinese Academy of Tropical Agricultural Sciences, Haikou 571101, Hainan, China Chinese Academy of Tropical Agricultural Sciences Hainan China; 2 School of Public Health, Key Laboratory of Tropical Translational Medicine of Ministry of Education, Hainan Medical University, Haikou 571199, Hainan, China Hainan Medical University Haikou China; 3 Chongzuo Key Laboratory for Conservation and Utilization of Edible and Medicinal Fungal Germplasm Resources, Guangxi Research Institute, Chinese Academy of Tropical Agricultural Sciences, Chongzuo 532100, Guangxi, China Chinese Academy of Tropical Agricultural Sciences Haikou China

**Keywords:** Inocybaceae, novel species, phylogeny, tropical China, Xizang

## Abstract

The species diversity of the ectomycorrhizal family Inocybaceae is poorly understood in tropical regions. The present work concerns a survey of macrofungi from Motuo in Xizang, which is located at the northern edge of China’s tropical zone. Three new species were identified using morphological features and DNA sequences (ITS, LSU and RPB2). *Inocybe flavitomentosa***sp. nov.** belongs to section Umbraticae. It has a yellow, densely hairy cap and angular spores with faint knobs. This is the first species in its group to be found in the Chinese tropics. *Inocybe motuoensis***sp. nov.** forms a basal branch in the *I. xanthomelas* group. It is the only purely tropical species in this group. It has a yellowish-brown cap, no caulocystidia and spores bearing 7–11 rounded nodules. *Inocybe subchondrospora***sp. nov.** belongs to the *I. fibrosoides* group. Resembling the northern species *I. chondrospora*, it has smaller spores (10.1–12.9 × 6.0–7.2 µm) and grows in subalpine areas at an elevation of 2,400–2,500 m. These discoveries expand the known range of *Inocybe* in tropical Asia and support the idea that the *I. xanthomelas* group likely originated in tropical regions.

## ﻿Introduction

The family Inocybaceae is a cosmopolitan group of ectomycorrhizal fungi, with approximately 1000 documented species worldwide. Tropical Africa or Tropical Asia was considered the ancestral area for the family (Matheny et al. 2009). However, the species diversity of the family remains relatively under-recognized in the Old World tropical regions (Horak 1979, 1980; Turnbull 1995; Horak et al. 2015; Gao et al. 2025a). Exploring the true species diversity of Inocybaceae in tropical regions is therefore important for understanding its evolutionary history.

*Inocybe* (Fr.) Fr. is the most species-diverse genus among the seven genera within the family. The genus is characterized by the presence of thick-walled pleurocystidia and mostly thick-walled cheilocystidia in the hymenial layer. Recent studies have established sectional clades within the morphologically defined sect. Marginatae using multigene phylogenies and morphological data (Matheny et al. 2022; Chen et al. 2024). However, the intricate nature of the genus and the existence of a large number of undescribed taxa complicate the exploration of its major evolutionary clades. This is particularly evident in tropical regions, where some species described with thin-walled cheilocystidia or even thin-walled pleurocystidia (Horak 1979; Gao et al. 2025a, b) are atypical for the genus.

The species diversity of *Inocybe* in tropical China has been poorly studied, with only a few new taxa recently described from Hainan and southern Yunnan Province (He et al. 2022; Gao et al. 2025a, b). Motuo, located in the southeastern Xizang Autonomous Region, harbors a tropical climate and represents the northernmost edge of China’s tropical region (http://www.motuo.gov.cn/). Although wood-inhabiting fungi have been studied in Xizang recently (Dai 2010; Cui et al. 2019; Dai et al. 2021; Wang et al. 2023, 2024; Yuan et al. 2023; Zhou et al. 2023; Qin et al. 2025; Zhou et al. 2025), the mycobiota of Motuo were previously less studied but have seen several research advances in recent publications (Song et al. 2022; Ma et al. 2023; Wu et al. 2022a, b, 2023; Wang et al. 2025; Zhu et al. 2022, 2024, Zhao et al. 2024; Cui et al. 2025a, b, Liu S et al. 2025; Liu ZB et al. 2025a, b). During our mycological expeditions in Motuo in 2023 and 2024, three new *Inocybe* taxa were identified and described based on molecular phylogeny and morphological studies.

## ﻿Materials and methods

### ﻿Specimen collection

Voucher specimens used in the present work were collected during 2023–2024 from Motuo, Xizang Autonomous Region of China. Field notes and digital photographs were made from fresh specimens. Fresh fruit bodies were dried in a portable electric oven at 45 °C overnight. The dried specimens were deposited in the Fungarium of the Institute of Tropical Bioscience and Biotechnology, Chinese Academy of Tropical Agricultural Sciences (**FCATAS**) and in the herbarium of Changbai Mountain Nature Reserve (**ANTU**) with FCAS number.

### ﻿Morphological study

The descriptions of macro-morphological characteristics were derived from field records and photographs. The size of each part of the basidiomata was recorded according to actual measurements in the field. The colors codes followed Kornerup and Wanscher (1978). Microscopic observations were carried out on tissue sections mounted in 5% KOH and a 1% aqueous solution of Congo red, and examined under an Olympus CX23 optical microscope. Lengths and widths of spores including nodules, cystidia excluding crystalline solids, and basidia excluding sterigmata were measured. For basidiospore descriptions, the notation (a)b–c–d(e) represents basidiospore dimensions, where the range b–d represented 90% of the measured values, a and e were the extreme values; Q indicated the length/width of the measured basidiospores, Qm referred to the average value, SD referred to the sample standard deviation, n/m/p means n basidiospores were measured from m basidiomata of p specimens (Hu et al. 2023; Wei et al. 2024).

### ﻿DNA extraction, PCR amplification, and sequencing

Total genomic DNA of basidiomata was extracted using an Ezup Column Fungi Genomic DNA Purification kit (Sangon Biotech, Shanghai, China) following the manufacturer’s instructions. Internal transcribed spacer (ITS), the nuclear large subunit ribosomal (LSU), and second largest subunit of RNA polymerase II (RPB2) were amplified and sequenced using primers pairs ITS1-F/ITS4 (Gardes and Bruns 1993), LR0R/LR7 (Vilgalys and Hester 1990), and bRPB2-6F/bRPB2-7.1R (Matheny 2005), respectively. PCR reactions were performed in a total volume of 25 μL containing 12.5 μL Taq PCR Master Mix (2, with Blue Dye), 9.5 μL ddH2O, 1 μL of each primer and 1 μL template DNA. Amplification reactions were performed with 1 min initial denaturation at 95 °C, followed by 35 cycles of denaturation at 95 °C for 30 s, annealing at 52 °C for 1 min, extension at 72 °C for 1 min, and a final extension at 72 °C for 8 min. The sequencing was performed by Sangon Biotech Co. (Shanghai, China) Ltd.

### ﻿Phylogenetic analyses

All newly generated sequences were submitted to GenBank, and BLASTn was searched on NCBI to select the closely related species for phylogenetic analyses. Tow sequences (CAL1310 and ZT9250) *Nothocybe distincta* (K.P.D. Latha & Manim.) Matheny and K.P.D. Latha were selected as outgroup.

The combined nuclear dataset (ITS+LSU+RPB2) was analyzed using Bayesian inference (BI) and maximum likelihood (ML). For ML, phylogenetic tree generation and bootstrap analyses were performed with the IQTREE Web Server (http://iqtree.cibiv.univie.ac.at/) using the ultrafast bootstrap analysis option and SH-aLRT branch testing, with bootstrap alignments set to 1,000, maximum iterations set to 1,000, and the minimum correlation set to 0.99 (Trifinopoulos et al. 2016). For BI, the best-fit model of evolution for each locus was selected using MrModeltest v2.3 (Nylander 2004), and BI analysis was performed using MrBayes 3.2.7 (Ronquist et al. 2012). The resulting phylogenies are visualized in FigTree v1.4.0 and modified and refined using the TVBOT online tool (https://www.chiplot.online/tvbot.html).

## ﻿Results

### ﻿Phylogenetic inference

As shown in Table 1, sequences of 72 taxa (96 ITS, 72 LSU, and 33 RPB2), including 20 new sequences (7 ITS, 7 LSU, and 6 RPB2) that were submitted to GenBank, were analyzed in this study. The final alignment comprised 2,770 nucleotides (790 ITS, 1300 LSU, and 680 RPB2) including gaps. For the BI analysis, the following substitution models were selected for each data partition using the AIC in MrModeltest v2.3: GTR+I+G for ITS and LSU, and GTR+G for RPB2. The BI phylogenetic analysis was stopped after 750,000 generations, when the average standard deviation of split frequencies converged to 0.009964, the effective sample size was 1965.73, and the average potential scale reduction factor parameter value was 1.002. The following models selected using the BIC were used for the ML analysis, which yielded a final log-likelihood value of −15901.732: TVM+F+I+G4 for ITS, K2P+I+G4 for LSU, and TNe+G4 for RPB2.

**Table 1.** A list of taxa used for the phylogenetic reconstruction. GenBank accession numbers, specimen numbers, origin and reference studies are given. Holotype specimens are labelled with HT, and lectotype specimens with LT. Species highlighted in bold were derived from this study. N/A: not available.

Species	Sample No.	Locality	Habitat	Genbank accession number			References
				ITS	LSU	RPB2	
*Inocybe alabamensis*				AY536280	N/A	N/A	Matheny 2005
* antoniniana *	KATO Fungi 4064 (HT)	Turkey	*Fagus sylvatica*	MN988712	N/A	N/A	Bandini et al. 2020
* bombina *	FR 0246007 (HT)			NR_173842	N/A	N/A	Bandini et al. 2019a
* castanea *	FYG1327	China	Coniferous forests	PP217760	PP230790	PP238459	Chen et al. 2024
* chondrospora *	M015162 (HT)			OR098441	N/A	N/A	Dovana et al. 2023
* decemgibbosa *	JV8115	Sweden		KT958905	KT958905	N/A	Vauras and Larsson 2015
* fibrosoides *	PAM01100301	Switzerland		HQ586857	HQ641098	N/A	Matheny and Wolfenbarger (unpublished)
*I. ailaoensis*	FYG2015385 (HT)	China	Fagaceae forests	PP217739	PP230780	PP238456	Chen et al. 2024
*I. alpinomarginata*	EL207-13	Sweden		MK153648	MK153648	N/A	Cripps et al. 2020
*I. angustifolia*	ZT10149	Thailand	*Castanopsis*, *Quercus*, *Lithocarpus*, and *Pinus*	GQ892990	GQ892944	N/A	Horak et al. 2015
*I. argenteolutea*	EL9906	Sweden		FN550889	FN550889	N/A	Ryberg et al. 2010
*I. bidumensis*	EL168 18	Sweden		OQ572784	OQ572784	N/A	Larsson (unpublished)
*I. blandula*	STU:SMNS-STU-F-0901577 (HT)	Austria	*Pinus sylvestris*	MZ144123	MZ144123	N/A	Bandini et al. 2021
*I. brevisquamulosa*	ZT10102 (HT)	Thailand	*Dipterocarpus obtusifolius* mixed	NR153123	NG057192	N/A	Horak et al. 2015
*I. calospora*	JFA 12539				AY038313	AY337365	Matheny et al. 2002
*I. caprimulgi*	JV10514F	Finland	*Betula*, *Pinus sylvestris*	KP308782	KP170953	N/A	Matheny and Bougher 2017
*I. corsica*	AH51900 (HT)	France		NR_176157	NG_088240	N/A	Crous et al. 2021
*I. dabaensis*	YZ2023102844 (HT)	China	Fagaceous trees and *Pinus*	PP993809	PP993852	PQ001020	Chen et al. 2024
*I. danxiaensis*	FYG4389 (HT)	China	Fagaceae forest	PP217735	PP230774	N/A	Chen et al. 2024
*I. diabolica*	JV5712F (HT)	Norway	Under *Betula pubescens* subsp.	HQ201350	N/A	N/A	Matheny and Wolfenbarger (unpublished)
*I. flavitomentosa*	XZ395 (HT)	China	tropical broad-leaved forests	PV823515	PV833587	PV845799	This study
*I. flavitomentosa*	XZ395A	China	tropical broad-leaved forests	PV823516	PV834863	PV847673	This study
*I. flavobrunnescens*	AH40466 (HT)	Portugal	*Quercus faginea*	KJ938784	N/A	N/A	Esteve-Raventós et al. 2015
*I. fuscobrunnea*	MR00378 (HT)	Burkina Faso	*Berlinia grandiflora*	MN096201	MN097893	MW219733	Aignon et al. 2022
*I. hirculus*	TURA 2577 (HT)	Finland		NR_153118	N/A	N/A	Vauras and Kokkonen 2009
*I. humilis*	G00126386 (HT)	Switzerland	* Picea *	ON227435	N/A	N/A	Esteve-Raventos et al. 2022
*I. invadens*	PERTH 08241716 (HT)	Australia	*Eucalyptus* or, *Corymbia calophylla*	NG057245	N/A	N/A	Matheny and Bougher 2017
*I. keteleeriicola*	FYG2884 (HT)	China	*Keteleeria* trees	PP217732	PP230769	PP238448	Chen et al. 2024
*I. krieglsteineri*	RFS031213-03 (HT)	Spain	*Pinus radiata*	KJ938768	N/A	N/A	Esteve-Raventós et al. 2015
*I. kuberae*	SMNS-STU-F-0901668	Germany		NR_184498	N/A	N/A	Bandini et al. 2022
*I. lacunarum*	EL-2015-JV12244			KT958908	KT958908	N/A	Larsson and Vauras 2015
*I. longistipitata*	LAH35274 (HT)	Pakistan		ON263121	N/A	N/A	Tan et al. 2022
*I. lutosa*	FYG2015298 (HT)	China	Fagaceous trees	PP993814	PP993851	N/A	Chen et al. 2024
*I. luxiensis*	FYG2857 (HT)	China	Fagaceae and Pinaceae forests	PP217733	PP230771	PP238450	Chen et al. 2024
*I. margaritispora*	AH40461	France		KT203785	N/A	N/A	Esteve-Raventós et al. 2015
*I. miyiensis*	HMJAU 24842 (HT)	China	Fagaceous trees	NR_153128	NR153128	N/A	Fan and Bau 2014
*I. motuoensis*	XZ1389 (HT)	China	tropical broad-leaved forests	PV827766	PV833643	PV845801	This study
*I. motuoensis*	XZ1389A	China	tropical broad-leaved forests	PV834867	PV834865	PV847675	This study
*I. obtusiuscula*	PAM02081710	France	Upper alpine with *Salix herbacea*	HQ586869	HQ641112	N/A	Matheny et al. (unpublished)
*I. ochracea*	Stangl-no-88 (HT)	Germany		NR_153119	N/A	N/A	Vauras and Kokkonen 2009
*I. olivaceonigra*	FYG2015350	China	Fagaceae forests	PP217746	PP230777	PP238454	Chen et al. 2024
*I. oreina*	JV15205	Sweden		OQ572790	OQ572790	N/A	Larsson (unpublished)
I. paludinella f. citrophylla	HRL0428	Canada		KX897418	N/A	N/A	Matheny et al. 2016 (unpublished)
*I. paludinelloides*	YGF2011143 (HT)	China	*Castanopsis* and *Pinus*	MG938541	MG825002	N/A	Chen et al. 2024
*I. parvisquamulosa*	TBGT12303 (HT)	India	On soil under *Aporosa* trees	NR185371	KT329453	N/A	Pradeep et al. 2016
*I. phaeocystidiosa*	AH9154 (HT)	Spain		KT203789	N/A	N/A	Esteve-Raventós et al. 2015
*I. phaeosticta*	PAM05091310	France		HQ586859	HQ641102	MH577435	Matheny et al. (unpublished)
*I. populea*	KT958911 (HT)			KT958911	N/A	N/A	Larsson and Vauras 2015
*I. populea*	TAKK15655			KT958911	N/A	N/A	Larsson and Vauras 2015
*I. praetervisoides*	AH29863	Spain		KT203794	N/A	N/A	Esteve-Raventós et al. 2015
*I. quercicola*	LAH (HT)	Pakistan		MW412768	N/A	N/A	Khan et al. 2022
*I. salicis*	DB1-6-12-2	Germany		MH366575	N/A	N/A	Bandini et al. 2019b
*I. simaoensis*	FYG2015395 (HT)	China	Fagaceous trees	PP217734	N/A	N/A	Chen et al. 2024
*I. similis*	SF14475	Italy	desert	MT704951	N/A	N/A	Dovana et al. 2020
*Inocybe* sp.	P09007	Korea		AB587748	N/A	N/A	Obase et al. 2011
*I. spectabilis*	FYG2342 (HT)	China	Birch forest mixed with *Populus davidiana*	PP217738	N/A	N/A	Chen et al. 2024
*I. spiniformis*	PBM 3748	Australia	*Allocasuarina torulosa* and other *Eucalyptus*	KP636868	N/A	N/A	Matheny and Bougher 2017
*I. strickeriana*	KR-KR-M-0044749	Germany		NR185399	NG228785	N/A	Bandini et al. 2019a
*I. subangustifolia*	RBI-794269 (HT)	Australia	*Eucalyptus* or *Acacia*	KP636870	NG057268	KM656112	Matheny and Bougher 2017
*I. subchondrospora*	XZ1230 (HT)	China	Mixed forests	PV827765	PV833642	PV845800	This study
*I. subchondrospora*	XZ1230A	China	Mixed forests	PV834866	PV834864	PV847674	This study
*I. subchondrospora*	NJ5283	China	Broad-leaved forests	PV834856	PV834860	NA	This study
*I. subrimosa*	3223 (LT)	Finland	Grass in a garden	ON227436	N/A	N/A	Esteve-Raventos et al. 2022
*I. substellata*	EL52-13	Sweden		KT958927	KT958927	N/A	Vauras and Larsson 2016
*I. tabacina*	PAM05071302	France		HQ586865	HQ641106	KM656115	Matheny et al. (unpublished)
*I. umbratica*	FYG251	China	Coniferous forests	PP217748	PP230778	N/A	Chen et al. 2024
*I. urbana*	AMB17142 (HT)			NR_138004	N/A	N/A	Franchi et al. 2015
*I. vaurasii*	AH47714 (HT)	France	*Fagus sylvatica*	ON263162	ON276736	N/A	Esteve-Raventos et al. 2022
*I. villosa*	KR-M 0042327 (HT)	Germany		MH366604	N/A	N/A	Bandini et al. 2019a
*I. xanthomelas*	G00127626 (LT)	France	Deciduous forest	ON227438	N/A	N/A	Esteve-Raventos et al. 2022
*I. xanthomelas*	AH47646	Spain	On calcareous soil	ON263161	ON276735	N/A	Esteve-Raventos et al. 2022
*Nothocybe distincta*	CAL1310	India		NR173156	NG057278	KX171345	Latha et al. 2016
*N. distincta*	ZT9250	India			EU604546	EU600904	Matheny et al. 2009

**Figure 1.**
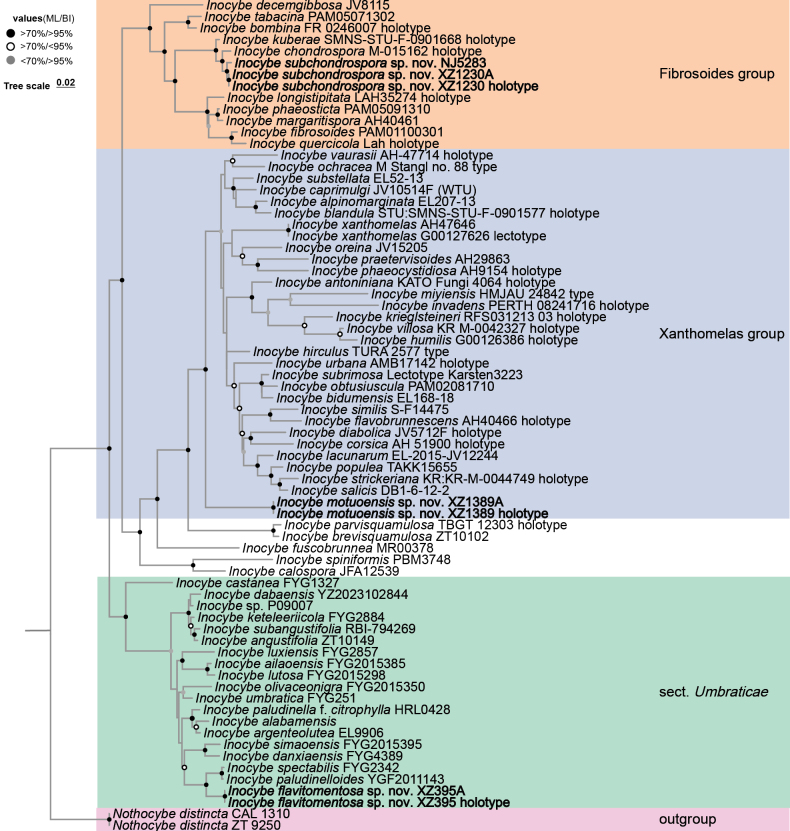
Phylogenetic tree of *Inocybe* based on the multigene alignment of ITS-LSU-RPB2 in the ML tree. ML bootstrap support (BS) ≥ 70% or Bayesian posterior probabilities (PP) ≥ 0.95 are indicated at the nodes. New species in this study are indicated in bold.

As shown in Fig. 1, three well-supported clades were retrieved in the three gene phylogeny, namely I. sect. Umbraticae, *I. fibrosoides* group, and the *I. xanthomelas* group. The three proposed new species nested in each of the three clades, separately, and formed independent lineages, indicating their unique phylogenetic positions. *Inocybe flavitomentosa* clustered with the lineage unifying two sister taxa (*I. spectabilis* and *I. paludinelloides*) sharing dull yellow basidiomata. *Inocybe subchondrospora* clustered with *I. chondrospora* Einhell. & Stangl in a fully supported lineage. *Inocybe motuoensis* is resolved as a basal lineage of the *I. xanthomelas* group. The two tropical Asian taxa, *I. parvisquamulosa* C.K. Pradeep & Matheny and *I. brevisquamulosa* E. Horak, Matheny & Desjardin, form a fully supported branch that constitutes a sister group to the *I. xanthomelas* group, while the West African taxon *I. fuscobrunnea* Aïgnon, Yorou & Ryberg forms a sister group to the aforementioned branch.

### ﻿Taxonomy

#### ﻿ Inocybe flavitomentosa

Taxon classificationFungiAgaricalesInocybaceae

A.H. Zhu, W.J. Yu, Hai X. Ma & X. Chen sp. nov.

77554266-9CC0-521F-8196-4D750A7001C1

859831

Figs 2, 3

##### Etymology.

Refer to the distinctive yellow and densely hairy (tomentose) surface of the mushroom’s pileus.

##### Holotype.

China • Xizang Autonomous Region: Linzhi City, Motuo County, in soil under tropical forest, alt. 1100 m, 21 July 2023, Hai X. Ma, FCATAS 9422 (XZ395). GenBank accession numbers: ITS (PV823515); LSU (PV833587); RPB2 (PV845799).

**Figure 2.**
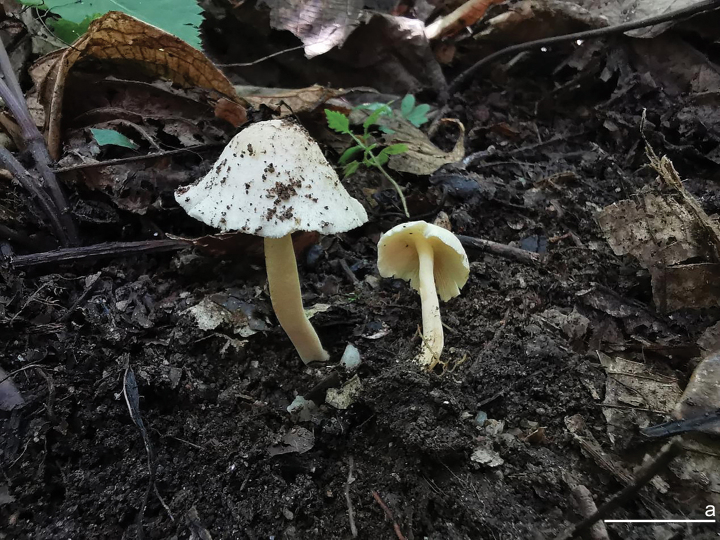
Basidiomata of *Inocybe flavitomentosa* (XZ395, holotype). Photos by Hai X. Ma. Scale bar: 10 mm.

##### Diagnosis.

*Inocybe flavitomentosa* is characterized by small to medium-sized basidiomata with yellowish white to pale yellow coloration, a subtomentose pileus surface, crowded lamellae, a slender entirely pruinose stipe, angular-nodulose basidiospores bearing faint apical knobs, and abundant lageniform to fusiform hymenial cystidia. While morphologically similar to *I. paludinelloides*, it differs from it by its smaller basidiospores with less pronounced knobs, smaller hymenial cystidia, and an ecology in tropical broad-leaved forests.

**Figure 3.**
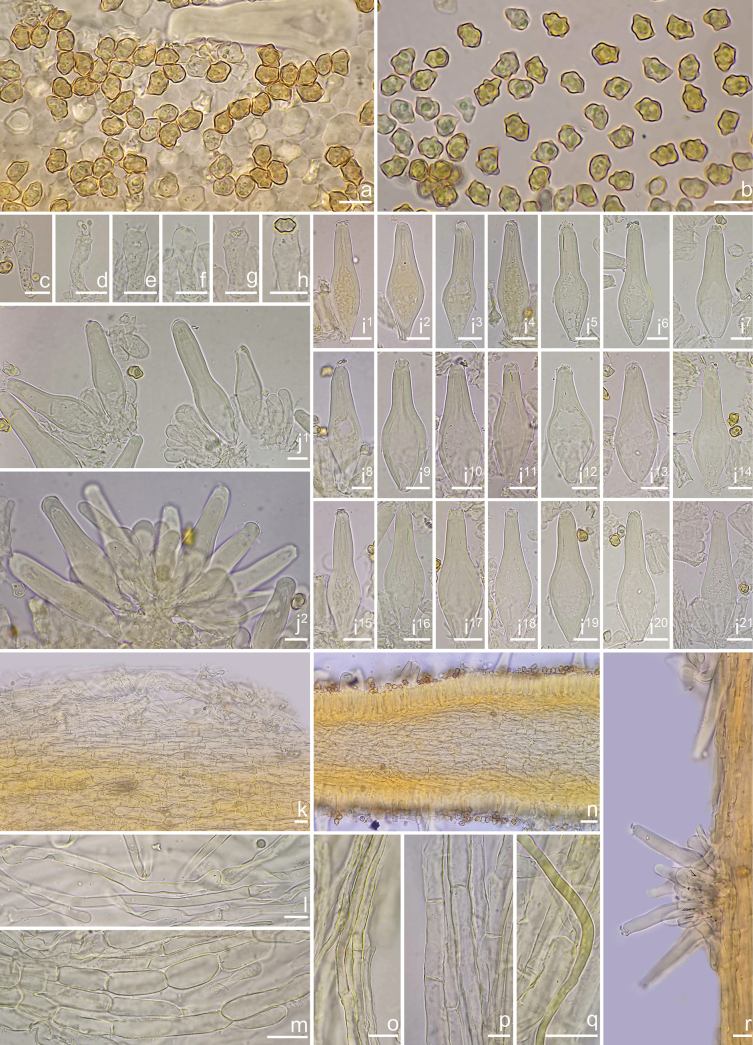
Microscopic features of *Inocybe flavitomentosa* (XZ395, holotype). a, b. Basidiospores; c–h. Basidia; i^1^–i^21^. Pleurocystidia; j^1^–j^2^. Cheilocystidia and cheiloparacystidia; k. Pileipellis; l. Pileipellis upper layer hyphae; m. Pileipellis lower layer layer hyphae; n. Cross-section of lamellae; o. Stipileipellis hyphae; p. Stipe trama hyphae; q. Oily hyphae; r. Caulocystidia on stipe surface. Photos by X. Chen. Scale bars: 10 μm (a–r).

##### Description.

***Basidiomata***: Small to medium-sized. ***Pileus*** 18–24 mm diam., hemispherical with decurved margin when young, becoming campanulate to parabolic, eventually broadly convex with a depressed to straight margin at maturity; surface dry, fibrillose to fibrillose-rimulose, finely subtomentose near margin; margin often splitting or strongly crenulate with age; coloration yellowish white (1A2) to pale yellow (2A2) or grayish white (2A1), darker toward the disc. ***Lamellae*** adnexed, crowded, alternating with 3–4 tiers of lamellulae, 0.8–1.5 mm wide; initially whitish yellow (1A2), maturing to pale yellow (2A3); edges entire to finely crenulate. ***Stipe*** 45–60 × 2.3–3.8 mm, central, solid, slightly swollen at apex and base; surface entirely white-pruinose (1A1), longitudinally striate, yellowish (2B3) to yellowish white (2A2). ***Context*** fleshy in pileus (0.5 mm thick at mid-radius, up to 2 mm under umbo), white (1A1); stipe context fibrous, yellowish white (2A2). Odor not recorded.

***Basidiospores*** [n=100/2/2] (6.3–)6.5–7.8(–8.1) × (4.3–)4.6–5.8(–6.0) μm, Q = (1.18–)1.20–1.65 (Q_m_ = 1.39 ± 0.09), angular-nodulose with 8–11 low nodules, pale yellowish to yellowish brown in 5% KOH; apiculus indistinct; occasionally containing 1 circular to oblong oil droplet. ***Basidia*** 15–30 × 4–8 μm, clavate to narrowly clavate or subcylindrical, predominantly 4-spored (rarely 2- or 3-spored). ***Pleurocystidia*** (n = 40) 48–73 × 12–21 μm, lageniform to fusiform or sublageniform, occasionally cylindrical; apices crystalliferous, obtuse; walls thick (up to 2.5 μm), colorless to pale yellow; abundant. ***Cheilocystidia*** (n = 40) 38–60 × 12–18 μm, morphologically similar to pleurocystidia but predominantly fusiform; walls <3 μm thick. ***Cheiloparacystidia*** 10–20 × 4–8 μm, clavate to elliptical, thin-walled. ***Caulocystidia*** (n=30) 35–74 × 7–14 μm, fusiform to lageniform or cylindrical, thick-walled (up to 3 μm), descending entire stipe. ***Cauloparacystidia*** 15–23 × 3–9 μm, clavate, thin-walled. ***Hymenophoral trama*** subregular, 40–75 μm thick; hyphae 3–12 μm wide, thin-walled. ***Pileipellis*** two-layered: upper layer (72–125 μm thick) of interwoven, rough, thin-walled hyphae (2–5 μm); lower layer of yellow to yellowish brown, subinflated hyphae (7–14 μm). ***Stipitipellis*** a cutis of pale-yellow cylindrical hyphae (2–5 μm). ***Oleiferous hyphae*** present in stipe trama (2–6 μm). Clamp connections ubiquitous.

##### Habitat and distribution.

Gregarious in humus-rich soil under tropical rainforests; currently known only from the type locality in southeastern Xizang.

##### Additional specimens examined.

China • Xizang Autonomous Region: Same locality as holotype, 21 July 2023, Hai-Xia Ma, FCATAS 14838 (XZ395A). GenBank accession numbers: ITS (PV823516); LSU (PV834863); RPB2 (PV847673).

##### Remarks.

*Inocybe flavitomentosa* is a dull yellow species phylogenetically sister to the lineage comprising *I. paludinelloides* T. Bau & Y.G. Fan and *I. spectabilis* Y.G. Fan, L.W. Qin & B. Wang. Although similar in yellow coloration, *I. paludinelloides* differs in having larger basidiospores, larger hymenial cystidia, and an ecological preference for mixed *Pinus-Castanopsis* forests in subtropical montane regions (Chen et al. 2024). In contrast, *I. spectabilis* possesses larger, elongate basidiospores with more pronounced nodules, larger hymenial cystidia, pyriform cheilocystidia, and is associated with *Betula* in northeastern China (Chen et al. 2024). Another yellow-tinged species, *I. danxiaensis*, is distinguished by a non-tomentose pileus, greenish-yellow stipe, fusiform hymenial cystidia that never form a neck, and rough epicutis hyphae (Chen et al. 2024). Finally, the Australian species *I. subangustifolia* Matheny, Bougher & Halling, though dull yellow, has fusiform hymenial cystidia, occurs on sandy soils under *Eucalyptus* or *Acacia*, and occupies a distinct phylogenetic position (Matheny and Bougher 2017).

#### ﻿ Inocybe motuoensis

Taxon classificationFungiAgaricalesInocybaceae

A.H. Zhu, W.J. Yu & Hai X. Ma sp. nov.

43F5B22C-9459-5FB9-B6C0-D35CA9F9F090

859832

Figs 4, 5

##### Etymology.

Named after Motuo County, the type locality in Xizang Autonomous Region, China.

##### Holotype.

China • Xizang Autonomous Region: Motuo County, Beibeng Town, in sandy soil under tropical forest, alt. 1100 m, 8 September 2024, Hai X. Ma & A.H. Zhu, FCATAS 14059 (XZ1389). GenBank accession numbers: ITS (PV827766); LSU (PV833643); RPB2 (PV845801).

**Figure 4.**
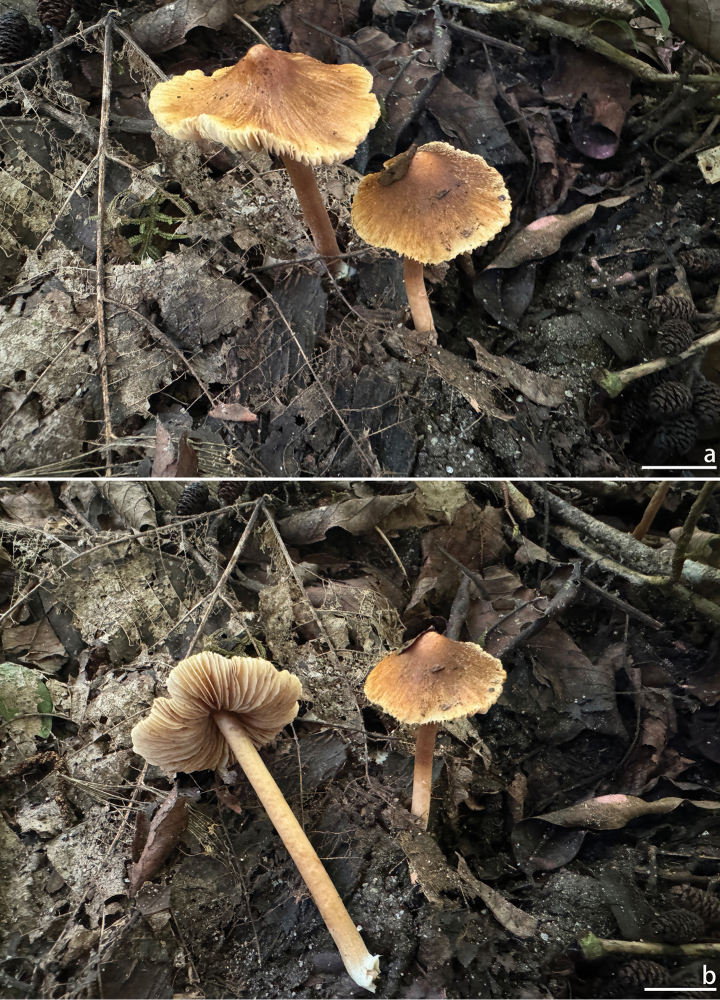
Basidiomata of *Inocybe motuoensis*. a, b. XZ1389 (holotype). Photos by A.H. Zhu. Scale bars: 10 mm (a, b).

##### Diagnosis.

Basidiomata small, Pileus yellowish brown with pallid margins; lamellae subcrowded; stipe glabrous, cylindrical, with a swollen base. Basidiospores nodulose with 7–11 distinct blunt nodules; hymenial cystidia fusiform with a tapered base and usually forming a pedicel, walls slightly thickened, yellowish, Caulocystidia absent. Occurs in broad-leaved forests in tropical Xizang, China. Most similar to I. humilis, but differs from it by the larger basidiospores with more pronounced nodules, mostly fusiform hymenial cystidia with thinner walls (1–1.5 µm thick), the lack of caulocystidia, and an ecology in tropical forest.

**Figure 5.**
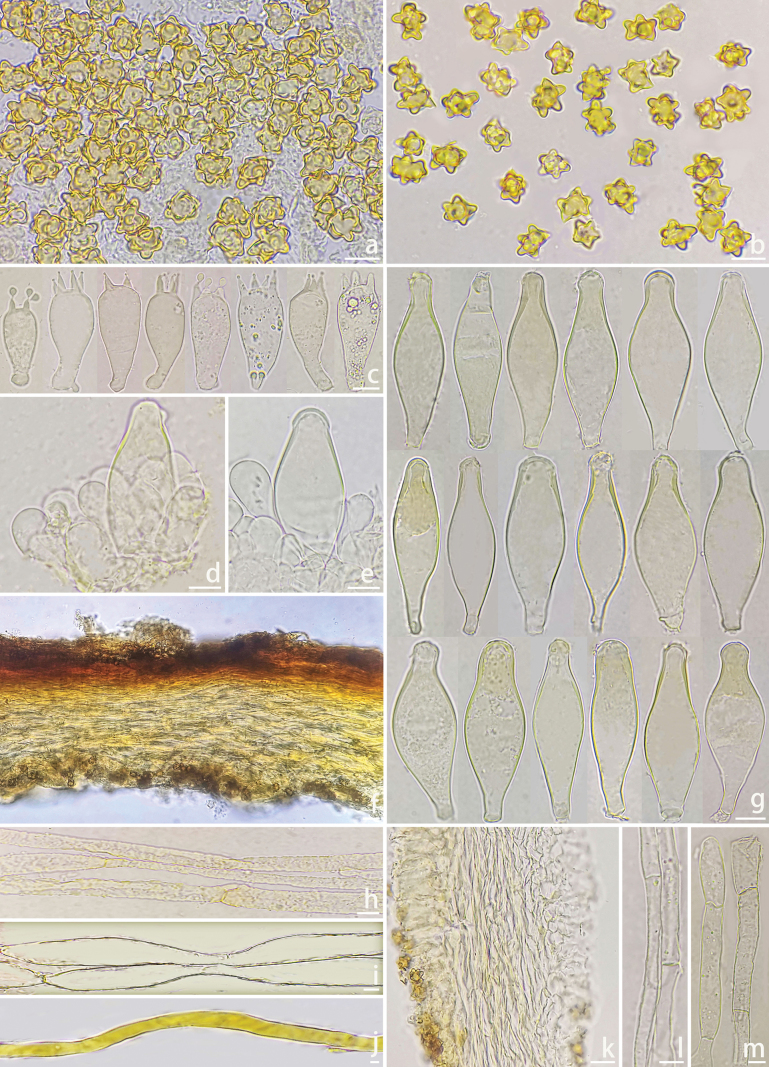
Microscopic features of *Inocybe motuoensis* (XZ1389, holotype). a, b. Basidiospores; c. Basidia; d, e. Cheilocystidia and cheiloparacystidia; f. Cross-section of pileus; g. Pleurocystidia; h. Pileipellis hyphae; i. Pileal tramra hyphae; j. Oily hyphae; k. Cross-section of lamellae; l. Stipitipellis hyphae; m. Stipe trama hyphae. Photos by J. Zheng. Scale bars: 10 μm (a–m).

##### Description.

***Basidioma*** small. ***Pileus*** 12–15 mm in diameter, campanulate to nearly conical when young, becoming plano-convex at maturity, with a distinct umbo at the center; margin even, spreading; surface dry, radially fibrillose with fine cracks to splitting, nearly smooth, sometimes with slightly raised small scales; center of the pileus brown (5B4) to umber brown (5C7), gradually fading towards the margin, mostly light brown (5B3) to pale yellowish-brown (6D5), margin pale yellow (1A3). ***Lamellae*** adnexed, subcrowded, 0.8–1.3 mm wide, unequal in length, with 3–4 tiers of lamellulae, edge faintly serrate; white (1A1) when young, becoming grayish-white (1B2), and at maturity pale yellow (1A3) to yellowish-brown (4B6). ***Stipe*** 20–30 mm long, 1.6–2.3 mm thick, cylindrical, solid; base swollen but non-marginate, up to 2.6 mm thick; glabrous throughout, covered with whitish (1A1) tomentose hyphae at the base; pale yellow (1A3), pale pinkish-brown (6C4) to yellowish-brown (6E7). ***Context*** of the pileus fleshy, brownish (5B3) near the surface, otherwise white (1A1); stipe context fibrous, brownish (6E5). Odor faintly salty.

***Spores*** [100/2/2] (8.1) 8.2–9.8–11.3 (12.1) × (6.5) 6.7–8.2–9.3 (10.1) µm, Q = (1.04) 1.06–1.20–1.40 (1.51), Qm ± Sd = 1.20 ± 0.1040, verrucose, with 7–11 distinct warts, yellow to yellowish-brown in 5% KOH. ***Basidia*** 27–38 × 8–15 µm, subcylindrical to clavate, bearing 2–4 sterigmata, sterigmata 4–9 µm long, occasionally up to 13 µm, thin-walled, hyaline, with internal oil droplets, sometimes translucent. ***Pleurocystidia*** 51–59–68 × 15.5–19.4–24 µm (n = 30), mostly fusiform, broadly fusiform, or narrowly fusiform, occasionally sublageniform, apex mostly obtuse, sometimes subcapitate, often encrusted, constricted at the base to form a short stalk, thick-walled, 1–1.5 µm thick towards the middle, with walls gradually thickening from the base upwards, thickest at the apex (up to 2–3 µm), walls distinctly yellow, mostly hyaline and lacking contents, occasionally with pale yellowish internal contents. ***Cheilocystidia*** 31–38–46 × 17.5–19.4–22 µm (n = 30), similar to pleurocystidia, fusiform to narrowly fusiform, with thick walls up to 2–3 µm at the apex, walls distinctly yellow. ***Cheiloparacystidia*** 20–28 × 8–13 µm, abundant, broadly clavate to obovate, thin-walled, hyaline, translucent. ***Hymenophoral trama*** 38–54 µm thick, subregular, composed of cylindrical to broadly cylindrical hyphae, cylindrical hyphae 5–7 µm wide, broadly cylindrical hyphae 13–25 µm wide. ***Pileipellis*** an epithelium, 30–60 µm thick, brown to brownish, composed of elongated cylindrical hyphae, 4–10 µm wide, thin-walled, surface rough, pale yellow. ***Pileus trama*** subregular, composed of inflated hyphae, 15–24 µm in diameter, relatively smooth, thin-walled, hyaline, and colorless. ***Stipitipellis*** composed of elongated cylindrical hyphae, 3–10 µm in diameter, pale yellow, mostly smooth, occasionally rough. ***Stipe trama*** hyaline, composed of narrow cylindrical and broadly cylindrical hyphae, narrow cylindrical hyphae 3–7 µm wide, broadly cylindrical hyphae 10–20 µm wide, thin-walled, smooth, and colorless. ***Oleiferous hyphae*** commonly observed in the stipe and pileipellis, 4–13 µm wide, smooth-surfaced, with protruding nodules, pale yellow to yellow. ***Clamp connections*** present in all parts of the basidioma.

##### Habitat & distribution.

Gregarious in humus-rich soil under tropical rainforests; currently known only from the type locality in southeastern Xizang.

##### Additional specimens examined.

China • Xizang Autonomous Region: Same locality as holotype, 8 September 2024, Hai X. Ma & A.H. Zhu, FCATAS 14835 (XZ1389A). GenBank accession numbers: ITS (PV834867); LSU (PV834865); RPB2 (PV847675).

##### Remarks.

*Inocybe motuoensis* is characterized by a yellowish-brown pileus, a glabrous stipe with a submarginatae base, basidiospores with conspicuous protruding rounded nodules, and slightly thick-walled hymenial cystidia. Phylogenetically, the new species belongs to the *I. xanthomelas* group but shows no clear affinity to other taxa within this group. The Xanthomelas group comprises species with small to medium-sized, slender basidiomata featuring yellow-ochre pilei, initially whitish to pale yellow stipe bearing a marginate basal bulb, and an indistinct odor. Diagnostic traits include elongated cystidia (high length-to-width ratio) and age-related darkening caused by intracellular pigments (yellow, ochraceous, or dark brown) within hymenial elements (cystidia, basidia) and contextual hyphae. These pigments intensify in aged or dehydrated specimens, resulting in brown-grey to near-black discoloration, with interspecific variation in pigment concentration (Esteve-Raventós et al. 2015). *Inocybe motuoensis* similarly exhibits darkening tissues due to pigmented cell walls in microstructures, consistent with the *I. xanthomelas* group. However, the absence of caulocystidia and presence of rounded nodules on basidiospores distinguish *I. motuoensis* within this species group.

#### ﻿ Inocybe subchondrospora

Taxon classificationFungiAgaricalesInocybaceae

A.H. Zhu, Hai X. Ma & Y.P. Ge sp. nov.

1DD21E82-34CC-561F-BFFD-AE1F8E466665

859834

Figs 6, 7

##### Etymology.

Refers to the basidiospore morphology which is similar to *I. chondrospora*.

##### Holotype.

China • Xizang Autonomous Region: Motuo County, Bangxin Town, along Motuo Highway, in sandy soil under conifer-broadleaf mixed forest, alt. 2,429 m, 13 June 2024, leg. Hai X. Ma & A.H. Zhu, FCATAS 12445 (XZ1230). GenBank accession numbers: ITS (PV827765); LSU (PV833642); RPB2 (PV845800).

**Figure 6.**
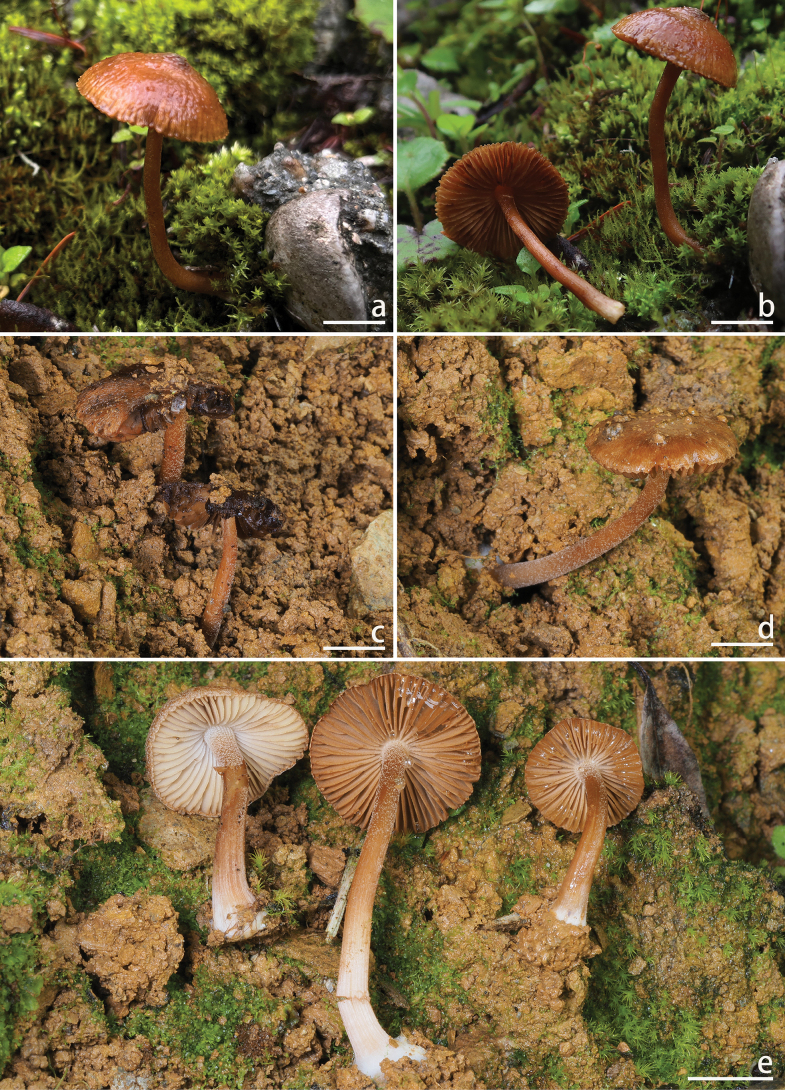
Basidiomata of *Inocybe subchondrospora*. a, b. XZ1230 (holotype); c–e. NJ5283. Photos by Hai X. Ma and Y.P. Ge. Scale bars: 10 mm (a–e).

##### Diagnosis.

Basidiomata small, Pileus brown to reddish brown; lamellae subcrowded; stipe cylindrical with a swollen base, brown to reddish brown, entirely pruinose. Basidiospores oblong-ellipsoid to phaseoliform, regular; hymenial cystidia broadly fusiform with a tapered base and usually forming a short pedicel, walls thickened, yellow, Caulocystidia descending to stipe base. Occurs in broad-leaved forests in tropical Xizang, China. Most similar to *I. chondrospora*, but differs from it by an appressed-fibrillose to nearly glabrous pileus, smaller basidiospores, broader hymenial cystidia, and an ecology in subtropical evergreen forests.

**Figure 7.**
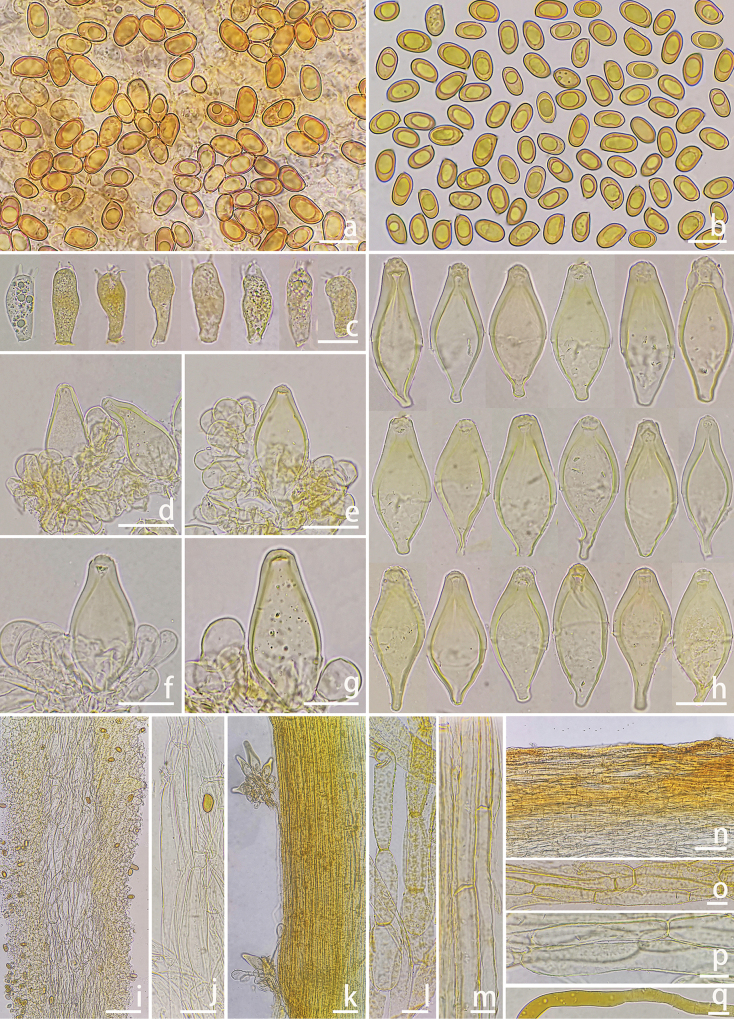
Microscopic features of *Inocybe subchondrospora* (XZ1230, holotype). a, b. Basidiospores; c. Basidia; d–g. Cheilocystidia and cheiloparacystidia; h. Pleurocystidia i. Cross-section of lamellae; j. Hymenophoral trama hyphae; k. Caulocystidia on stipileipellis hyphae; l. Stipileipellis hyphae; m. Stipe trama hyphae; n. Pileipellis; o. Pleipellis hyphae; p. Stipe trama hyphae; q. Oily hyphae. Photos by Y.R. Xu. Scale bars: 10 μm (a–c, j, l–m, o–q); 20 μm (d–h); 50 μm (i, k, n).

##### Description.

***Basidiomata*** small-sized. ***Pileus*** 15–20 mm diam., initially hemispherical to subglobose, becoming convex to plano-convex with age, developing a low obtuse umbo at maturity; margin persistently incurved in young specimens, decurved in age; surface dry, matte, radially striate near margin; disc umber (7B6) to chestnut brown (6E4), gradually fading to yellowish brown (5A5–6A6) towards margin. ***Lamellae*** adnexed, close, with 3–4 tiers of lamellulae; lamellar face brownish yellow (5D6) when dry, developing sparse dark brown (5E4) maculations; edges fimbriate, concolorous with faces. ***Stipe*** 15–19 × 2–3 mm, eccentric, terete, solid; surface densely white-pruinose (1A1) at apex, becoming fibrillose-striate downwards; apex strong brown (6E7) with punctate protrusions, gradually transitioning to brown (6B6) at base. ***Context*** fleshy in pileus, 2–3 mm thick at dis, fibrillose in stipe; yellowish white overall, developing brownish tones near stipe base. Odor not distinctive.

***Basidiospores*** [100/2/2] (holotype) mostly ellipsoid to oblong-ellipsoid, (9.5)10.1–11.4–12.9(13.4) × (5.9)6.0–6.6–7.2(8.1) μm, Q = (1.3)1.5–1.7–1.9(2.0), Q_m_ ± SD = 1.73 ± 0.134; less often phaseoliform, 9.5–10.9–11.9 × 5.9–6.5–8.1 μm; smooth, yellow-greenish in KOH, containing 1–2 yellowish refractive oil droplets. ***Basidia*** 14–18–22 × 6–7–9 μm (n=30), predominantly clavate to cylindrical, 4-sterigmate (occasionally 2-sterigmate); sterigmata 2–8 μm long; contents yellowish with granular inclusions. ***Pleurocystidia*** 41–49–59 × 17–21–27 μm (n=30), fusoid-ventricose with broad basal pedicels (3–5 μm wide), walls thickened apically (2–4 μm), yellow-green in KOH. ***Cheilocystidia*** 40–46–60 × 11–22–25 μm (n = 30), morphologically similar to pleurocystidia. ***Cheiloparacystidia*** 9–14–20 × 8–11–15 μm (n = 30), clavate, thin-walled, hyaline. ***Hymenophoral trama*** regular, 38–72 μm wide; hyphae cylindrical to inflated (7–30 μm diam.), thin-walled, yellowish. ***Pileipellis*** 48–130 μm thick, cutis-type; hyphae parallel, 7–24 μm diam., walls yellowish brown with encrusted pigments. ***Stipe trama*** composed of parallel, cylindrical hyphae 6–14 μm diam. Caulocystidia 38–42–47 × 14–17–20 μm (n = 30), fusoid, thick-walled (2–3 μm), concentrated at stipe apex. ***Oleiferous hyphae*** present in all tissues, 3–12 μm diam., bright yellow, irregularly contorted. ***Clamp connections*** abundant throughout all tissues.

##### Habitat and distribution.

Gregarious in sandy soils of subalpine zone (2,400–2,500 m elev.); currently known only from the type locality in southeastern Xizang, also found in subtropical evergreen broad-leaved forests in Hubei province.

##### Additional specimens examined.

China • Xizang Autonomous Region: Motuo County, Bangxin Town, along Motuo Highway, in sandy soil under conifer-broadleaf mixed forest, alt. 2,429 m, 13 June 2024, leg. Hai X. Ma & A.H. Zhu, FCATAS 14836 (XZ1230A); GenBank accession numbers: ITS (PV834866); LSU (PV834864); RPB2 (PV847674) • Hubei Province: Yichang City, Wufeng Tujia Autonomous County, 17 June 2024, in subtropical evergreen forests dominated by fagaceous trees, Y.P. Ge, Q. Na, J.W. Guo, G.Y. Qiu & L.J. Wang, NJ5283 (FCAS4254). GenBank accession numbers: ITS (PV834856); LSU (PV834860).

##### Remarks.

*Inocybe subchondrospora* is distinguished by small, reddish-brown to cinnamon-brown basidiomata, a convex to plano-convex pileus, and pruinose stipes with a swollen but non-marginate base. Microscopically, it exhibits ellipsoid to elongate basidiospores with obtuse apices and broadly fusiform hymenial cystidia bearing small pedicels. Phylogenetically, *I. subchondrospora* forms a sister lineage to the north temperate taxon *I. chondrospora* (syn. *I. immigrans* Malloch), with this clade itself sister to *I. kuberae* Bandini & B. Oertel.

*Inocybe chondrospora* resembles the new species in basidiomata coloration and spore shape but differs in having a tomentose-scaly pileus, significantly larger basidiospores, larger hymenial cystidia (pleurocystidia: 38–80 × 11–28 µm), and an ecological preference for sandy soils associated with Betulaceae, Orchidaceae, Pinaceae, and Salicaceae (Dovana et al. 2023). In contrast, *I. kuberae* shares the dark brown pileus and pruinose stipe but diverges in nodulose spores (avg. 11.8 × 8.9 µm), ventricose (sub)lageniform hymenial cystidia, and occurrence on calcareous soils at subalpine elevations (Bandini et al. 2022).

## ﻿Discussions

This study describes three new *Inocybe* species collected from Motuo, Xizang, enriching our understanding of fungal diversity in the tropical climatic zone of the Tibetan Plateau. Motuo lies within the collision zone between the Indian and Eurasian Plates, traversed by the Yarlung Zangbo Suture. This region was once part of the Paleo-Tethys Ocean, and the gorges formed by plate compression and uplift served as refugia for Tertiary paleotropical flora. An elevation gradient spanning 7,000 meters – from 200 m (Basika) to 7,782 m (Namcha Barwa) – creates nine vertical climatic zones, driving rapid species diversification over short distances. Among the three new species, *Inocybe flavitomentosa* and *I. motuoensis* are currently known only from their type localities in tropical monsoon forests. In contrast, *I. subchondrospora* also occurs in subtropical forests of Hubei Province; in Motuo, it inhabits subtropical forests above 1,100 m, suggesting biogeographic links between this region and the subtropical vegetation zone of Central China.

In the three-gene phylogeny reconstructed herein, *Inocybe flavitomentosa* belongs to sect. Umbraticae and is closely related to *I. spectabilis* and *I. paludinelloides*, which similarly exhibit yellow-toned basidiomata. Sect. Umbraticae was recently segregated from sect. Marginatae (Chen et al. 2024), characterized by small basidiomata (white, grayish-white, or yellow), densely pruinose stipes, nodulose-angular spores, abundant thick-walled hymenial cystidia, and a two-layered pileipellis. Morphologically, *I. flavitomentosa* aligns well with the core features of sect. Umbraticae. Previously, only *I. angustifolia* (Corner & E. Horak) Garrido was known from tropical Asia in this section, with most species distributed in subtropical regions of Southwest China. The discovery of *I. flavitomentosa* extends the range of sect. Umbraticae into China’s tropical climatic zone.

*Inocybe motuoensis* clusters within the *I. xanthomelas* group in the three-gene phylogeny, with *I. motuoensis* positioned in a basal branch. Notably, *I. motuoensis* is the only tropical species in this group. Additionally, *I. parvisquamulosa* and *I. brevisquamulosa* from tropical Asia (Horak et al. 2015) and the recently described West African *I. fuscobrunnea* (Aïgnon et al. 2022) show close affinity to the *I. xanthomelas* group. Although the tropical Asian species possess distinctly smaller basidiomata (Horak et al. 2015), differing from the core morphology of the *I. xanthomelas* group, their phylogenetic placement implies a tropical origin for this lineage.

*Inocybe subchondrospora* resides within the *I. fibrosoides* group and is sister to the boreal species *I. chondrospora*. Interestingly, while the *I. fibrosoides* group typically exhibits medium-sized basidiomata with distinctly nodulose-angular spores, both *I. chondrospora* and *I. subchondrospora* possess predominantly smooth, ellipsoid spores (Dovana et al. 2023), occasionally with irregular forms – a significant deviation from other group members. Similarly, *I. similis* Bres. (phylogenetically nested in the *I. xanthomelas* group) displays mainly smooth spores with rare weak angular projections (Dovana et al. 2023). Ancestral state reconstruction by Ryberg et al. (2010) indicated that smooth (phaseoliform) spores represent the most probable ancestral state in *Inocybe* s.l. (>80% probability), with nodulose spores being derived in *Inocybe* s.str. yet subject to multiple independent reversal events. The discovery and phylogenetic placement of *I. subchondrospora* further underscore the complex evolutionary trajectory of spore morphology in *Inocybe*.

## Supplementary Material

XML Treatment for Inocybe flavitomentosa

XML Treatment for Inocybe motuoensis

XML Treatment for Inocybe subchondrospora

## ﻿Additional information

### Use of AI

No use of AI was reported.

### Author ORCIDs

An-Hong Zhu https://orcid.org/0000-0002-2812-8108

Yan-Ru Xu https://orcid.org/0009-0004-8382-1636

Hai-Xia Ma https://orcid.org/0000-0001-6699-7454

Wen-Jie Yu https://orcid.org/0000-0002-0147-3091

### Data availability

Publicly available datasets were analyzed in this study. All newly generated sequences were deposited in GenBank (https://www.ncbi.nlm.nih.gov/genbank/; accessed on 27 June 2025; Table 1). All new taxa were deposited in MycoBank (https://www.mycobank.org/; accessed on 2 July 2024; MycoBank identifiers follow new taxa).
